# Nursing interventions for rehabilitation in Parkinson's disease: cross mapping of terms

**DOI:** 10.1590/1518-8345.0689.2728

**Published:** 2016-08-08

**Authors:** Michelle Hyczy de Siqueira Tosin, Débora Moraes Campos, Leonardo Tadeu de Andrade, Beatriz Guitton Renaud Baptista de Oliveira, Rosimere Ferreira Santana

**Affiliations:** 1 RN, MSN, Centro Internacional SARAH de Neuroreabilitação e Neurociências, Rio de Janeiro, RJ, Brazil. Master's Student, Escola de Enfermagem Aurora de Afonso Costa, Universidade Federal Fluminense, Niterói, RJ, Brazil.; 2 RN, Centro Internacional SARAH de Neuroreabilitação e Neurociências, Rio de Janeiro, RJ, Brazil. Master's Student, Escola de Enfermagem Aurora de Afonso Costa, Universidade Federal Fluminense, Niterói, RJ, Brazil.; 3 RN, MSN, Hospital SARAH Belo Horizonte, Belo Horizonte, BH, Brazil. Doctoral Student, Universidade Federal de Minas Gerais, Belo Horizonte, BH, Brazil.; 4 PhD, Full Professor, Departamento de Fundamentos de Enfermagem e Administração, Universidade Federal Fluminense, Niterói, RJ, Brazil.; 5 PhD, Adjunct Professor, Departamento de Enfermagem Médico-Cirúrgico, Universidade Federal Fluminense, Niterói, RJ, Brazil.

**Keywords:** Nursing Process, Classification, Rehabilitation, Parkinson Disease

## Abstract

**Objective:**

to perform a cross-term mapping of nursing language in the patient record with the Nursing Interventions Classification system, in rehabilitation patients with Parkinson's disease.

**Method:**

a documentary research study to perform cross mapping. A probabilistic, simple random sample composed of 67 records of patients with Parkinson's disease who participated in a rehabilitation program, between March of 2009 and April of 2013. The research was conducted in three stages, in which the nursing terms were mapped to natural language and crossed with the Nursing Interventions Classification.

**Results:**

a total of 1,077 standard interventions that, after crossing with the taxonomy and refinement performed by the experts, resulted in 32 interventions equivalent to the Nursing Interventions Classification (NIC) system. The NICs, "Education: The process of the disease.", "Contract with the patient", and "Facilitation of Learning" were present in 100% of the records. For these interventions, 40 activities were described, representing 13 activities by intervention.

**Conclusion:**

the cross mapping allowed for the identification of corresponding terms with the nursing interventions used every day in rehabilitation nursing, and compared them to the Nursing Interventions Classification.

## Introduction

In recognition of the first descriptions performed by James Parkinson in 1817, Parkinson's disease (PD) was thus entitled years later by Jean-Martin Charcot. The mean age of onset of symptoms is 60 years of age, and the incidence increases with age and may affect up to 5% of the population over 79 years of age. The mean duration of the disease, from diagnosis through death, is 15 years, and the relationship of mortality in males to females is two to one^1^. 

The causal relationships remain as evasive as when described in 1817, however, pathological signs related with genetic and environmental components are strongly discussed ^1^ ^-^ ^2^. Moreover, radical changes in the conceptualization of the disease, starting with a better understanding of the motor and non-motor pathological manifestations, by means of understanding that the neurodegenerative process can begin even before the onset of motor symptoms, enabled scientific advances in its treatment^2^. Currently, medications and non-pharmacological therapies aim at alleviating the symptoms and improving the quality of life of this population.

In this sense, the nursing care provided to the individual with this disease, permeates through the aspects of this condition related to symptomatology; it is considered to be multisystemic, progressive, and incurable. In the rehabilitation context, the nurse, as a professional member of the multidisciplinary team, has an important role in health promotion, treatment of complications, and adaptation to the limitations imposed by the disease. The nurse directs the plan of care to meet the needs of each patient and family, guiding the search for the patient's independence in relation to his/her physical, cognitive and behavioral limits through an appreciation of his/her potential^3^.

In this scenario, the adoption of standardized nursing care prevails, based on legal, ethical, scientific and methodological premises. Thus, care grounded in the nursing process enables interactivity, since it is based on mutual relationships of nurses, the multidisciplinary team, the patient and the family^3^. Beyond the completion of the nursing process steps, based on the use of a language classification system, the universality of information is ensured, which provides the dissemination of concepts and the practical applicability of interventions^4^ ^-^ ^5^.

The Nursing Interventions Classification (NIC) is among the various classification systems referring to the interventions to be used with the nursing process. 

This taxonomy, of North American origin and worldwide scope, was developed in order to document and communicate nursing care by integration of data in computer systems, and provides a source of data for research^6^ ^-^ ^7^. According to this classification system, a nursing intervention is defined as "any treatment, based upon clinical judgment and clinical knowledge, that a nurse performs to enhance patient/client outcomes"^6^.

Therefore, the importance of studies that address the nursing interventions in the rehabilitation of patients with PD, structured in an elected classification system, is undeniable. The nursing research that addresses this issue converges on the current global trend to better understand the disease, its symptoms and treatment, contributing to the scientific progress in this area, which will be reflected in improved patient care. However, currently, it is observed that nursing scientific production in this context remains scarce^8^ ^-^ ^11^. 

Based on the above, this study aimed to perform the nursing language term cross-mapping of the records of rehabilitation patients with Parkinson's disease, with the Nursing Interventions Classification (NIC) system.

## Method

This was a descriptive, quantitative study, developed in accordance with the technical procedure of documentary research, using records as a primary source of data collection. The methodological framework uses concepts and principles of cross-mapping. This method was chosen because it allows the linguistic comparison and semantics between non-standardized terminologies with the chosen classification system^4^ ^,^ ^7^ ^,^ ^12^.

The International Center for Neurorehabilitation and Neuroscience in the city of Rio de Janeiro, Brazil, was the scenario for this research. Children and adults with neurological sequelae, originating from congenital or acquired central nervous system injury, receive care in the rehabilitation area of this center. The team is interdisciplinary and patient care includes contextualized and individualized guidance, recommending the involvement of family members and caregivers.

This center has electronic medical records of patients in which the care provided by the nurse is documented without the use of standardized language. For this research, the authors considered the records containing nursing interventions described as nursing terms in natural language.

From the inauguration of this Centre, in March 2009, until the beginning of this research, in April 2013, there were 1,266 patients admitted who were diagnosed with PD: 796 had nursing assessments. To determine the sample, the inclusion criteria included those patients whose chart contained five or more nursing records, totaling 167. The records of patients who, in addition to the diagnosis of PD, had a medical diagnosis that characterized other parkinsonian syndromes, such as secondary Parkinsonism, for example, were excluded. Thus, 148 records were obtained. From this total, the calculation for a probability sample of the simple random type was calculated using the formula^13^: 



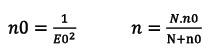



In this case: N = 148 records (population size), E = 9% (tolerable sampling error), n0 = 123 records (first approximation of the sample size) and n = 67 records (sample size).

Thus, the sample consisted of 67 patient charts, which represented 45% of the total population, and 9% of sampling error. Of these, the last five nursing assessments were considered for this study, totaling 335 analyzed nursing assessments..

The study limitation is the adoption of a 9% error, instead of 5% that is usually used as maximum error. However, this limitation was considered by the multiplicity of nursing records obtained in each medical chart, which provided significant data collection for the researchers.

After conducting three pilot tests, for training and improvement of three researchers on the method, the cross-mapping was completed, simultaneously, from June to December of 2013, in three stages: 1) extraction and standardization of terms, 2) separation and comparison of non-standardized terms with those standardized by the NIC, and 3) evaluation and refinement of the mapping.

The first stage was conducted from August to November of 2013, in which the three researchers performed the electronic extraction of information that comprised the database containing: 1) patient data, 2) medical diagnosis and the progression of PD, 3) parts of nursing assessment containing the context of the intervention, and 4) separation of the nursing language terms that indicated or excluded intervention hypotheses (verbs). For example; in the part of the nursing evaluation, which was described "Performing bladder re-education", the term "bladder re-education" was highlighted after the fragmentation of the record. Similarly, in the part in which there was the description, "The patient was instructed regarding home adaptation strategies to promote greater safety for the performance of daily activities", the words: "Instructed" "Home adaptation" and " Safety" were highlighted in the database.

Data were arranged in an Excel spreadsheet for Windows, and then, normalized according to the adequacy of verbal tenses, spelling correction, standardization of gender and number, and the exclusion of repetitions, synonyms and casual expressions that do not designate particular concepts.

From December of 2013 to May of 2014, three researchers proceeded to the second stage through cross-mapping of the terms identified in the previous step, with the NIC taxonomy. The following rules of cross-mapping were considered: 1- mapping the "meaning" of the words, not just the words, 2- using the "keyword" in the intervention to map to the NIC intervention, 3- using verbs as "keywords" in the intervention, 4-mapping the intervention from the NIC intervention label for the activity, 5- maintaining consistency between the intervention being mapped and the NIC definition of the intervention, 6- using the more specific NIC intervention label and 7- mapping interventions that have two or more verbs for the two or more corresponding NIC interventions^12^.

The nursing terms in natural language were compared with the NIC interventions. The categorization of the nursing terms was performed with a combination of analyses, where: when the term found, was matched with the classification system term, it was categorized as an exact match, and when that term had similar concepts, synonyms and related terms, it was categorized as a partial match. For presentation of the results, the exact and partial matches were considered to have the same value.

In the examples previously cited, the term "bladder reeducation", extracted after the fragmentation of parts of the nursing record, and assumed to be a nursing intervention, was considered to be an exact match with NIC. However, the terms "oriented", " home adaptation "and "safety" were considered to be partial matches, and were correlated to the standardized NIC intervention, "Environmental Management: Safety." In this case, a consistency was observed between the context of the intervention described in the patient record, with the definition of the intervention proposed by NIC, which is: "Monitoring and manipulation of the physical environment to promote safety"^6^.

The data of this stage were organized under the title of the intervention, definition, non-standardized terms and standard NIC terms that corresponded to confirmatory clinical evidence for the presence of the intervention.

From June of 2014, the third stage was achieved by means of the evaluation and refinement of cross-mapping. At this stage, expert nurses analyzed the collected data; two were experts in nursing classification and three in the PD rehabilitation. For selection of these nurses, a minimum clinical experience of five years, or a doctorate with experience in research on nursing classification systems was required. The relationship between the contexts of interventions, the non-standardized terms and NIC interventions was established through the agreement of experts. This step was conducted in individual cycles and in-group. In the second cycle, the consensus of experts was obtained, and the statistical agreement analysis was not necessary.

Data were analyzed considering their absolute frequency, percentage, means and standard deviations. The development of the study met the national and international standards of ethics in research involving human subjects (Protocol No 691310).

## Results 

The characteristic profile of the 67 patients of the sample was: 63% male, mean age of 69.3 (± 10). The progression of PD ranged from one to 24 years, with a higher representation of those who had one to eight years of disease progression (75%).

In the case of nursing interventions, there were1,077 standard interventions identified in the 67 patient charts analyzed, representing a mean of 16 interventions per patient. The highest concentration of interventions was focused on the context of health promotion. After cross-mapping with the taxonomy, and refinement made by the experts, 32 interventions equivalent to the NIC terminology emerged. Of these, nine (28%) had a frequency of occurrence higher than 50%. The interventions, "Teaching: Disease Process," "Patient Contracting," " Learning Facilitation," and "Teaching: Group" were present in all patient charts, with a mean of 2.6 repetitions. This occurred because nurses in different contexts implemented these interventions. (Table 1)

**Table 1:** Distribution of nursing interventions, according to the Nursing Interventions Classification - NIC, present in 67 records of patients with Parkinson's disease. Rio de Janeiro, RJ, Brazil, 2014

NIC code: Nursing intervention	n	%
5602: Teaching: Disease Process	67	100
4420: Patient Contracting	67	100
5520: Learning Facilitation	67	100
5604: Teaching: Group	67	100
0430: Bowel Management	64	96
0440: Bowel Training	64	96
6486: Environmental Management: Safety	44	66
5246: Nutritional Counseling	40	60
0590: Urinary Elimination Management	34	51
2380: Medication Management	31	46
0570: Urinary Bladder Training	27	40
4120: Fluid Management	27	40
4046: Cardiac Care: Rehabilitative	17	25
7820: Specimen Management	7	10
4040: Cardiac Care	6	9
5100: Socialization Enhancement	6	9
4390: Milieu Therapy	6	9
7110: Family Involvement Promotion	5	7
1800: Self-Care Assistance	5	7
2314: Medication Administration: Intravenous (IV)	4	6
1860: Swallowing Therapy	4	6
0582: Urinary Catheterization: Intermittent	2	3
7140: Family Support	1	1
1280: Weight Reduction Assistance	1	1
7110: Family Involvement Promotion	1	1
1260: Weight Management	1	1
3584: Skin Care: Topical Treatments	1	1
0610: Urinary Incontinence Care	1	1
5230: Coping Enhancement	1	1
7120: Family Mobilization	1	1
3590: Skin Surveillance	1	1
0224: Exercise Therapy: Joint Mobility	1	1

During the analysis of the patient records, it was possible to identify that nurses performed all steps of the nursing process. With regard to interventions, these were performed in two ways: interventions of an individual character and interventions conducted with a group of patients.

For individual assistance, the nurse performs consultations based on the nursing process, guided by clinical reasoning. However, even without the adoption of a standardized language, the nursing diagnoses, interventions and expected outcomes are established, which are described in the patient records. In the planning step, the nurse evaluates whether the result to be achieved requires intervention by means of individual or group approach. However, guided by clinical reasoning many patients were included by nurses for both approaches,. In other words, for those patients who had not achieved the expected outcomes, these were also individually addressed after group intervention, and vice versa.

The data shown in Table 2 demonstrate that nine nursing interventions equivalent to the NIC classification system, were prescribed concurrently at individual (19%) and group (54%) levels, representing 73% of the total number of interventions.

**Table 2:** Categorization of nursing interventions equivalent with the Nursing Interventions Classification - NIC, which were repeated in the records. Rio de Janeiro, RJ, Brazil, 2014

NIC code: Nursing interventions	Group		Individual	
	n	%	n	%
5602: Teaching: Disease Process	67	100	26	39
4420: Patient Contracting	67	100	26	39
5520: Learning Facilitation	67	100	26	39
0430: Bowel Management	36	54	28	42
0440: Bowel Training	41	61	23	34
6486:Environmental Management: Safety	11	16	33	49
5246: Nutritional Counseling	13	19	27	40
2380: Medication Management	24	36	7	10
4046: Cardiac Care: Rehabilitative	6	9	11	16

**Figure 1:**
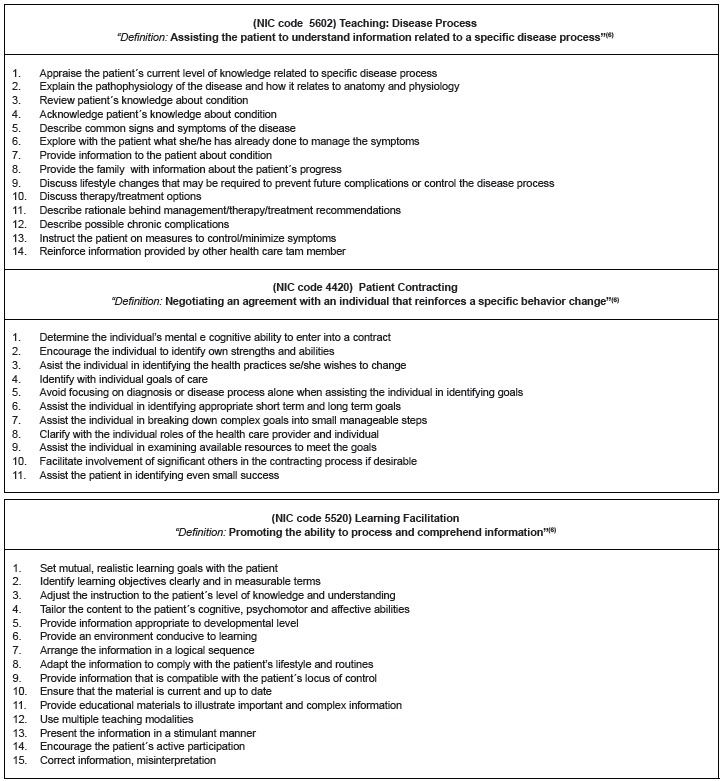
Activities implemented in accordance with the prescribed nursing interventions and matched to the Nursing Interventions Classification - NIC. Rio de Janeiro, RJ, Brazil, 2014

In addition, the standardization of the approaches with patient groups was identified, represented by lectures, interactive sessions, and providing illustrative teaching materials. The topics of the lectures focused on the interrelationship of the pathophysiological aspects, pharmaco-therapeutics and lifestyle, in the context of rehabilitation. Thus, the theme of each class was focused on: the PD; the risk factors for cerebrovascular diseases, such as hypertension, diabetes mellitus and dyslipidemia; bowel constipation; and, activities of daily living. For all of these, Figure 1 shows in a descriptive manner, the primary interventions implemented by nurses, with their respective activities, in order to better analyze and understand the role of the rehabilitation nurse. A variety of activities were demonstrated (total of 40), representing 13 activities implemented by prescribed intervention.

## Discussion

In terms of characteristics of the sample of this research, the findings converge with studies showing that PD tends to occur more frequently in men, especially in the age group over 60 years^1^.

Regarding the progression of the disease, the results of this study have representativeness, showing that the neurodegenerative process of PD is nonlinear, but it is concerned with individual aspects^14^. However, the decompensation rate is much faster in the early stage of the disease, leading to functional impairment of the patient that should be evaluated in order to consider his/her personal characteristics^14^. In addition, patients in the early stages of the disease can demonstrate more doubts and anxieties about this disease. This requires that the rehabilitation nurse takes a more careful look at the educational aspects, giving information about current symptoms to these patients, considering those of a prognostic nature^3^. Therefore, nurses who provide care to patients with PD should consider the magnitude of aspects, and their interventions should respect the peculiarities inherent to the individual process of the disease progression.

With regard to nursing interventions, the results demonstrate effectiveness of the methodological tools used in this study, which enabled the achievement of objectives. The cross-mapping identified nursing language terms prescribed by nurses in the records of patients with PD who participated in the rehabilitation program, and compared them to the standardized NIC language, which is globally recognized. This method is a viable tool in the standard language implementation process in health services, as it allows the nurses to compare data consistently and generalizable^7^ ^,^ ^12^. 

Furthermore, nursing interventions, as part of the nursing process, are recognized in the care plan, which is developed in order to eliminate or minimize a nursing diagnosis, seeking to achieve the goal or pre-established outcome^4^. 

Thus, the interventions mapped and described in this study are highlighted, which were directly linked to educational practice, and used by nurses as the main tool for health promotion. Health promotion, as a change strategy in technical healthcare models, has been used for decades as an alternative for expanding the quality of health and life of the population, by intervention with individuals and the understanding of the health/disease process, such as social production^15^.

In the context of neurological rehabilitation, the actions for health promotion are aimed toward recovery, but, mainly, for the adaptation to limitations imposed by the disability, according the needs of each patient/family. These actions are primarily guided by functional, motor, psychosocial and spiritual aspects^15^. Nurses must establish a bond with the patient/family and guarantee guidance for the health/disease process, providing the necessary resources for facilitation and implementation of this learning. The autonomy of the individual is important within this relationship, asserting the principles of citizenship and democracy, socially committed to improving health status in accordance with the principles of neurorehabilitation^15^.

The nursing interventions related to intestinal disorders, which in this study were representatively described, lead to research showing that among the non-motors symptoms of PD, bowel constipation is the most prevalent. This alteration is present in 70-80% of these individuals, and is mainly related to neuronal degeneration occurring in the vagal center of bowel control, and which may be present at any stage of neurodegeneration^16^. In this scenario, nurses action aim to intervene to restore bowel function of patients with non-pharmacological measures that minimize neurological damage, due to the degenerative process.

However, these measures depend on change in the patient's lifestyle. The nurses use references beyond the biological in their work methodology, and recognize that the actions required for adherence to long-term treatment and care are deeply interrelated with the culture, i.e., with the lifestyle, habits, routines and rituals in the lives of patients, converging with contemporary scientific references^17^. The concepts described seek complicity with the patient/family for active participation in the process of change and adaptation to achieve outcomes. In this context, when the nurse identifies a failure in achieving the outcome proposed through non-pharmacological therapy, a discussion with the medical team on the need for pharmacologic intervention may be needed for treatment of intestinal disorders. Thus, the primary interprofessional collaboration for rehabilitation is established^3^. 

In the analysis of the activities developed, educational actions based on contextual understanding to guide the individual / family are carried out according to the main interventions. However, a multiplicity of alternatives and the creativity of nurses involved are evident in the rehabilitation process of the patient with PD, which by means of generalized actions, attempts to address the peculiarities inherent to each individual/family. They seek to empower patients to work effectively on their social reintegration. This provides a dynamic movement and permanent redefinition of knowledge for acquiring skills and attitudes that are better for a quality of life, by acquisition of a critical-reflexive attitude^16^.

The application of the nursing process in the practice of neurorehabilitation is evidenced by the results of this study, through the expression of the clinical method used by nurses in the rehabilitation of patients with PD. The performance of the following specific steps of the nursing process (collection of multidimensional information on health status, identification of conditions that require nursing interventions, planning the necessary interventions, implementation and evaluation of actions) provided the nursing care to the individual / family, in order to consider their singularities, and in an extended mode, converge with professional recommendations widely discussed in the nursing literature^18^. 

During the analysis of records, the recognition of the nurses about their role in the rehabilitation process with the patient who progresses with a neurodegenerative, multisystem and still incurable disease was clear.. Their full involvement in this process allowed for prescribing interventions, mostly permeated with the principles of health promotion. Thus, rehabilitation was based on the search for patient potential, aimed at his/her restoration through reciprocal and realistic goals. These actions may lead to the opportunity for a life with better quality, reemergence of self-esteem, independence and family involvement, which confirms the principles of neurorehabilitation, as discussed in the scientific community area^16^.

## Conclusion

The cross-mapping enabled the comparison of existing information in the patient records of patients with PD with the standardized interventions of the NIC. In addition, the nursing interventions used in the clinical practice of rehabilitation nurses were identified, with actions grounded in health promotion and family involvement.

Standardization of language is encouraged to enable documentation of nursing information, contributions to patient care and facilitation of communication between nurses and other health professionals. The use of the NIC terminology can contribute to standardizing nursing care within the rehabilitation of patients with PD, in order to ensure quality of the professional care, leading to significant benefits for the profession.

The existing gaps in scientific knowledge about the effectiveness of nursing interventions in this population constitute the main limitation of this study, since there was no possibility of such comparative data due to the lack of studies in this area. Furthermore, further studies are needed, with more representative sample sizes, to minimize the possibility of error.
